# Multi-vessel Versus Culprit-vessel-only PCI for STEMI: Where Does the Jury Stand?

**DOI:** 10.1016/j.amsu.2021.102343

**Published:** 2021-04-24

**Authors:** Talal Almas, Ayesha Akram, Maryam Ehtesham, Reema Ahmed, Tarek Khedro, Uzair Malik, Lamees Alshaikh, Lina Alshaikh, Yasar Sattar, Hafeez Ul Hassan Virk

**Affiliations:** a RCSI University of Medicine and Health Sciences, Dublin, Ireland; b Rawalpindi Medical University, Rawalpindi, Pakistan; c Icahn School of Medicine at Mount Sinai, NY, USA; d Harrington Heart & Vascular Institute, Case Western Reserve University, University Hospitals Cleveland Medical Center, Cleveland, OH, USA

The annual incidence of myocardial infarctions (MI) in the United States is 1.5 million. Percutaneous coronary intervention (PCI) is a non-surgical procedure whereby a stent is employed to revascularize a blocked coronary vessel. It has become the preferred modality for the treatment of acute ST-segment elevation myocardial infarction (STEMI) [1,2]. In patients presenting with acute coronary syndrome (ACS), the first question that governs the optimal treatment approach is whether there is an obvious culprit lesion for the patient's underlying presentation. If such a lesion is present alongside ongoing ischemia in a STEMI, emergent PCI of the culprit vessel is recommended. Contrarily, if the culprit lesion is present without ongoing ischemia, the extent of coronary artery disease (CAD) must be evaluated to determine the approach: a single-vessel (PCI of culprit) or multivessel (including the left coronary artery) PCI. The culprit lesion is treated first; however, in patients with multiple coronary artery disease, the distal lesions are treated first. Nevertheless, whether multivessel PCI confers a comparative therapeutic advantage over culprit-vessel-only PCI remains enigmatic.

In 2013, the American College of Cardiology (ACC)/American Heart Association (AHA) discouraged primary PCI of non-culprit vessels in STEMI patients who are hemodynamically stable (class III: harm) [3]. Patients suffering from cardiogenic shock were the only exception in whom emergent revascularization of non-culprit stenoses could be considered, though no evidence substantiating this recommendation was found. Following trials that suggested potential benefit in early non-culprit PCI in stable patients with STEMI, the guidelines were updated to recommend the use of culprit-vessel PCI (CV–PCI) for hemodynamically stable patients. In addition, the guidelines on multivessel PCI (MV-PCI) were updated from class III to class IIb such that it is now recommended for hemodynamically stable patients with multivessel disease during the primary PCI or later as a planned, staged procedure [4]. Although the 10.13039/501100000860European Society of Cardiology (ESC) is in corroboration with the ACC/AHA guidelines regarding CV-PCI, there is stronger class IIa evidence supporting MV-PCI during the initial procedure when the STEMI is accompanied by cardiogenic shock or as a planned staged procedure for patients with multivessel disease [5]. Given the fact that 50% of patients presenting with STEMI also have a multivessel disease, MV-PCI and CV-PCI have remained at the epicenter of a cardiology conundrum [5].

Although it has been previously reported that more major adverse cardiovascular events (MACE) are associated with MV-PCI, a meta-analysis published in 2020 comparing the risks of MACE as well as procedural outcomes of MV-PCI and CV-PCI reported inconclusive findings for cardiovascular mortality, all-cause mortality, and procedural complications [6]. The data reported strongly suggests that MV-PCI confers benefit over CV-PCI in patients with a STEMI on a background of multivessel disease. Therefore, MV-PCI should be the first-line treatment for this group. Furthermore, in a review of 10 different metanalyses regarding the use of MV-PCI vs CV-PCI, we conclude that in patients with STEMI, MV-PCI is more efficacious than CV-PCI. A common advantage found among the studies was the reduction in the rates of both revascularization and non-fatal reinfarction with the use of MV-PCI. Additionally, a decrease in MACE was appreciated in the majority of the cases [[6], [7], [8], [9], [10], [11], [12], [13], [14], [15]]. Some of the studies demonstrated no difference in all-cause mortality [[10], [11], [12], [13]]. These findings are delineated in Table 1 below.

**Table 1:** Comparative disease outcomes in patients undergoing either multivessel or culprit-only PCI.

	Sample size	Type of PCI	Risk of repeat revascularization	Major adverse cardiac event (MACE)	Outcome
Levett JY^6^	6751 patients	MV-PCI	Lower rate (3.7% vs 12.3%; RR: 0.33; 95%CI: 0.25 to 0.44)	Reduction in the rate of MACE (13.1% vs 22.1%; RR: 0.54; 95%CI: 0.43 to 0.66)	Inconclusive findings for all-cause mortality, cardiovascular mortality, and procedural complications
		CV- PCI	Higher rate		–
Garcia DC^7^	1044 patients	MV-PCI	Lower rate (RR 0.38, 95% CI 0.27–0.53, P < 0.00001)	–	Reduced incidence of non-fatal reinfarctions
		CV- PCI	Higher rate	–	–
Feistritzer HJ^8^	6314 patients	MV-PCI	Lower rate (HR 0.33, 95% CI 0.22–0.50, *p* < 0.001)	–	Reduced incidence of non-fatal reinfarctions
		CV- PCI	Higher rate	–	–
Osman M^9^	7423 patients	MV-PCI	Lower rate (4.0% vs 11.7%, RR 0.44, 95% CI 0.28 to 0.70, p < 0.0001)	Significant reduction in the rate of MACE (10.7% vs 18.6%, RR: 0.64, 95% CI 0.51 to 0.81, p = 0.002)	–
		CV- PCI	Higher rate	–	–
Atti V^10^	7030 patients	MV-PCI	Lower rate (RR: 0.34; 95% CI: 0.25 to 0.44)	–	Lower risk for reinfarction No difference in all-cause mortality
		CV- PCI	Higher rate	–	–
Rai D^11^	6930 patients	MV-PCI	Lower rate (RR 0.36, 95% CI 0.25–0.53, *P* < 0.0001)	Reduction in the rate of MACE (RR 0.58, 95% CI 0.46–0.72, *P* < 0.0001)	Lower risk for reinfarction No difference in all-cause mortality
		CV- PCI	Higher rate	–	–
Dahal K^12^	840 patients	MV-PCI	Lower rate (RR = 0.35, 0.24–0.52, P < 0.00001)	Reduction in the rate of MACE (RR = 0.46, 95% CI: 0.35–0.60, P < 0.00001)	Lower risk for reinfarction No significant difference in all-cause mortality
		CV- PCI	Higher rate	–	–
Vaidya SR^13^	2991 patients	MV-PCI	Lower rate (RR = 0.38, 95% CI = 0.30–0.47; P < 0.00001)	Reduction in the rate of MACE (RR = 0.54, 95% CI = 0.41–0.71; P < 0.00001)	No benefit on all-cause mortality and nonfatal MI.
		CV- PCI	Higher rate	–	–
Villablanca PA^14^	2006 patients	MV-PCI	Lower rate (OR, 0.39; 95% CI, 0.30–0.51)	Reduction in the rate of MACE (OR, 0.62; 95% CI, 0.43–0.90)	Lower risk for reinfarction No differences observed between MV versus CV-PCI for subsequent MI
		CV- PCI	Higher rate	–	–
Politi L^15^	263 patients	MV-PCI	Lower rate	Reduction in the rate of MACE (23.1% vs 50.0%	Better patient outcome with reduction of non-fatal reinfarction rate
		CV- PCI	Higher rate	Highest rate of long-term MACE	–

**Legend**. PCI: percutaneous coronary intervention; MV-PCI: Multi-vessel PCI; MACE: Major adverse cardiac event; MI: Myocardial Infarction.

In patients with ACS, a key determinant of whether MV-PCI should be performed during the initial procedure depends on the evidence of cardiogenic shock. According to the CULPRIT-SHOCK trial, if there is cardiogenic shock, only the culprit lesion should be treated [16]. If not, simultaneous or staged PCI of other lesions should be considered. The trial reported that culprit lesion-only PCI for patients presenting with cardiogenic shock and evidence of multivessel disease on angiography was associated with better outcomes, less all-cause mortality or renal replacement therapy (RRT) at 30 days (45.9%) compared to patients who had an immediate MV-PCI (55.4%) (hazard ratio [HR] 0.83, 95% confidence interval [CI] 0.71–0.96, p = 0.01).

Additionally, the COMPLETE trial, a randomized, multi-center trial, yields mounting evidence towards the success of MV-PCI in patients with STEMI and multivessel CAD after initial successful culprit-only PCI [17]. A total of 2025 patients were stratified to receive culprit-lesion-only PCI and guideline-directed medical therapy, and another 2016 received complete revascularization in addition to guideline-directed medical therapy. At a median 3-year follow-up, the primary outcomes determined were: (1) composite of cardiovascular (CV) death or new MI or (2) CV death, new MI, or ischemia-driven revascularization (IDR). The results demonstrated that staged MV-PCI reduced MACE in patients with STEMI and CAD [17,18].

Despite its ostensible benefits, single-procedure MV-PCI requires the employment of a higher dose of contrast and radiation in comparison with staged MV-PCI and therefore accentuates the risk of operator-patient fatigue [19]. In patients with complex lesions, it is in most cases optimal to stop and have a full discussion with the patient or family. The optimal treatment modality also depends on whether the site in which the diagnostic angiogram is performed is capable of undergoing PCI, especially in cases requiring complex PCI. In fact, a complicated structural anatomy, such as chronic total occlusion, is the most common anatomical abnormality that can preclude the uptake of PCI [20]. The perplexity of this decision is further compounded when comorbidities such as prior MI and diabetes are present. These considerations need to be taken into account carefully to determine if PCI can be performed safely [20].

In most studies comparing MV-PCI with CV-PCI in the context of STEMI, the clinical benefit conferred by MV-PCI compared to CV-PCI has largely been established. At the same time, however, this superiority may not be the result of complete revascularization itself but of better baseline anatomy and comorbidities compared to the patients who did not undergo complete revascularization. Contrarily, there is less convincing data in patients with NSTEMI and patients with stable ischemic disease. This notion needs to be explored through the means of further trials to better inform the debate on what constitutes the most optimal treatment.

We presume the data we have is sufficient but might be part of a larger picture yet to be fully elucidated!

## Funding

Na.

## Ethical approval

Na.

## Consent

Na.

## Author contribution

TA, AK, ME: conceived the idea, designed the study, and drafted the manuscript. RA, TK, UM: conducted literature search and created the illustrations. LA, LA,: revised the manuscript critically and refined the illustrations. YS, HUHV: revised the final version of the manuscript critically and gave the final approval.

## Registration of research studies

1. Name of the registry: NA.

2. Unique Identifying number or registration ID: NA.

3. Hyperlink to your specific registration (must be publicly accessible and will be checked): NA.

## Guarantor

The Guarantor is the one or more people who accept full responsibility for the work and/or the conduct of the study, had access to the data, and controlled the decision to publish.

Talal Almas.

## Declaration of competing interest

Na.
